# Parallel Computation of EM Backscattering from Large Three-Dimensional Sea Surface with CUDA

**DOI:** 10.3390/s18113656

**Published:** 2018-10-28

**Authors:** Longxiang Linghu, Jiaji Wu, Zhensen Wu, Xiaobing Wang

**Affiliations:** 1 School of Physical and Optoelectronic Engineering, Xidian University, Xi’an 710071, China; lxlinghu@stu.xidian.edu.cn; 2 School of Electronic Engineering, Xidian University, Xi’an 710071, China; wujj@mail.xidian.edu.cn; 3 Collaborative Innovation Center of Information Sensing and Understanding, Xidian University, Xi’an 710071, China; 4 Science and Technology on Electromagnetic Scattering Laboratory, Shanghai 200438, China; iswangb@163.com

**Keywords:** electromagnetic backscattering, large three-dimensional sea surface, normalize radar cross section, graphic processing units (GPUs), compute unified device architecture (CUDA)

## Abstract

An efficient parallel computation using graphics processing units (GPUs) is developed for studying the electromagnetic (EM) backscattering characteristics from a large three-dimensional sea surface. A slope-deterministic composite scattering model (SDCSM), which combines the quasi-specular scattering of Kirchhoff Approximation (KA) and Bragg scattering of the two-scale model (TSM), is utilized to calculate the normalized radar cross section (NRCS in dB) of the sea surface. However, with the improvement of the radar resolution, there will be millions of triangular facets on the large sea surface which make the computation of NRCS time-consuming and inefficient. In this paper, the feasibility of using NVIDIA Tesla K80 GPU with four compute unified device architecture (CUDA) optimization strategies to improve the calculation efficiency of EM backscattering from a large sea surface is verified. The whole GPU-accelerated SDCSM calculation takes full advantage of coalesced memory access, constant memory, fast math compiler options, and asynchronous data transfer. The impact of block size and the number of registers per thread is analyzed to further improve the computation speed. A significant speedup of 748.26x can be obtained utilizing a single GPU for the GPU-based SDCSM implemented compared with the CPU-based counterpart performing on the Intel(R) Core(TM) i5-3450.

## 1. Introduction

Studying the electromagnetic (EM) backscattering characteristics from an electrically large sea surface plays an important role in the synthetic aperture radar (SAR) imaging, ocean parameter inversion, targets detection, and monitoring [1,2,3]. Computing the normalized radar cross section (NRCS) constitutes a prerequisite to investigate on characteristics of backscattering echoes from large sea surfaces. Based on the two-scale oceanic surface model simulated by the double superimposition model (DSM) [4,5], the Kirchhoff approximation (KA) [6,7], small perturbation method (SPM) [8], small-slope approximation method (SSA) [9,10], two-scale method [11], four-modified two-scale method (FMTSM) [12], and slope-deterministic composite scattering model (SDCSM) [13] can be quoted to calculate the NRCS from sea surface. The KA method is only valid to the calculation of EM scattering when the incident angle closes to the specular direction (±20°). SPM, TSM, and FMTSM are suitable for the calculation of the Bragg scattering from sea surface. However, when the incident angle is small, these methods will not be adequate to calculate the specular scattering from the sea surface. Due to the insuperable computational complexity, the SSA method is not suitable to deal with EM scattering echoes from an electrically large sea surface. The SDCSM, which combines the quasi-specular scattering of Kirchhoff Approximation (KA) and Bragg scattering of the two-scale model (TSM), has been successfully utilized to calculate NRCS from large oceanic surface [14,15]. However, with the development of SAR, the resolution of radar is gradually improved which makes the calculation of NRCS from an electrically large sea surface time-consuming and inefficient. It is very necessary to develop an efficient method to improve the calculation speed of NRCS based on SDCSM. 

Nowadays, graphics processing units (GPUs) are specialized for compute-intensive, highly parallel scientific problems due to their multi-threaded, many-core processor with tremendous computational horsepower and very high memory bandwidth [16]. In 2006, compute unified device architecture (CUDA) was introduced by NVIDIA as a general purpose parallel technology to make it easy for programmers to utilize GPU for many complex computational problems [17,18]. For scientists and engineers, GPUs with CUDA have been an attractive alternative to conventional CPU counterparts which can improve the performance of massive scientific computing problems. In [5], the NVIDIA Tesla K80 GPU with five CUDA optimization strategies has been utilized to speed up the generation of the time-evolving oceanic surface model (TOSM) and a significant 791.0× speedup has been achieved compared to a serial C program. A GPU-based vector radiative transfer (VRT) algorithm for vegetation electromagnetic scattering has been designed and achieved a speedup of 213-fold on the NVIDIA Fermi GTX480 [19]. Wu et al. [20] proposed a GPU-parallel optimization for image autoregressive interpolation and to achieve a high 147.3 times speedup compared to single-threaded C program. A CUDA-based implementation in calculating large sea surface EM scattering based on the SSA method was developed in [21,22]. In [23], GPU has been utilized for parallel computation of the reflected radiation from an aerial target and a speedup of 426× has been achieved on the NIVIDIA K20c GPU. GPU has been also utilized for weather and research forecast (WRF) and the effects of diverse optimization strategies on the performance were discussed in detail. The processing time reduced to 43.5 ms executed on GPU compared to 16,928 ms executed on CPU [24]. 

In this paper, a new GPU-based SDCSM implemented with five CUDA optimization strategies is developed for calculating the NRCS from an electrically large sea surface. This paper is organized as follows: first, based on the electrically large oceanic surface simulated by double superimposition model (DSM) [4,5], the SDCSM is exploited to calculate the NRCS and the results are compared with the experimental data to verify the correctness of the outcomes. Next, an initial GPU-based SDCSM implemented is proposed based on CUDA. Then, finally, five CUDA strategies are adopted to further improve the computing performance and the effects of block sizes and number of registers per thread on computing performance is analyzed. After using diverse CUDA strategies, a significant speedup can be achieved by the GPU-based SDCSM algorithm executed on Tesla K80 compared with a serial C program executed on Intel(R) Core (TM) i5-3450 CPU. 

## 2. Electromagnetic Backscattering from an Electrically Large Sea Surface

**Figure 1:**
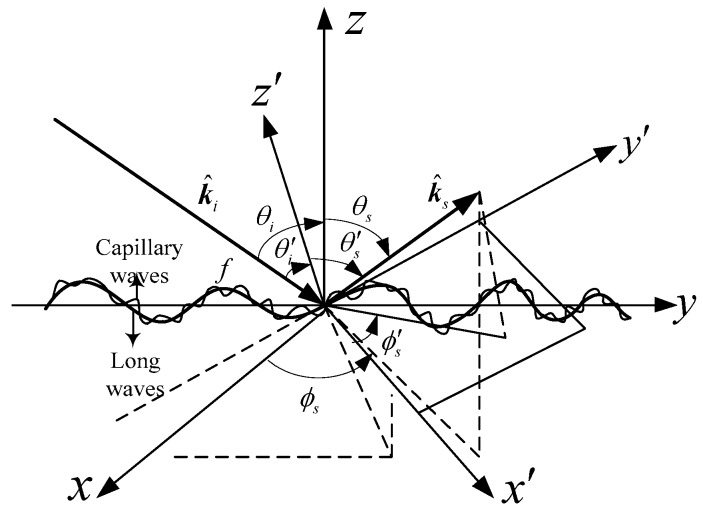
Illustration of electromagnetic (EM) scattering from an electrically large oceanic surface.

The conventional oceanic surface is generally divided into two scales: the large-scale gravity waves and small-scale ripples superimposed on them, where the large-scale gravity can be simulated as millions of triangular meshing. In order to calculate the EM backscattering echoes from diverse triangular meshing on the electrically large sea surface, a hypothesis should be followed that the EM backscattering echoes from diverse facets on the oceanic surface are de-correlated [25]. In this section, a slope-deterministic composite scattering model (SDCSM) is developed to calculate the NRCS of EM backscattering from the electrically large sea surface taking both scattering mechanisms into account, as shown in Figure 1.

### 2.1. Slope-Deterministic Kirchhoff Approximation Model (SDKAM)

When the incident angle is small ($\theta_{i}\leq 20{}^{\circ}$), the quasi-specular scattering mechanism corresponding to large-scale gravity waves plays a leading component for the EM backscattering echoes from the electrically large sea surface. Thus the Kirchhoff approximation method (KAM) can be utilized to calculate the NRCS, which can be given as [6,7]

(1) $$\sigma_{pq}^{KAM}({\hat{k}}_{i},{\hat{k}}_{s})=\pi k^{2}{\left|\vec{q}\right|}^{2}{\left|{\tilde{F}}_{pq}^{KAM}\right|}^{2}Prob(Z_{x},Z_{y})/q_{z}^{4}\;$$

where $\vec{q}=k({\hat{k}}_{s}-{\hat{k}}_{i})=[q_{x},q_{y},q_{z}]$, *p* and *q* represent radar reception and incident polarizations, respectively; ${\tilde{F}}_{pq}^{KAM}$ denotes the coefficient matrix depending on the incident angles $\left(\theta_{i},\;\phi_{i}\right)$, scattering angles $\left(\theta_{s},\;\phi_{s}\right)$, and Fresnel coefficients [7]. $Prob(z_{x},z_{y})$ denotes the slope probability density function of the gravity waves; $z_{x}=-q_{x}/q_{z}$, $z_{y}=-q_{y}/q_{z}$. The NRCS of the EM backscattering can be well calculated by the KAM when the incident angles close to the quasi-specular direction (±20°). 

### 2.2. Slope-Deterministic Two-Scale Model (SDTSM)

As the incident angle increases, EM backscattering echoes from the electrically large sea surface are dominated by the Bragg scattering mechanism caused by small-scale ripples. In this case, The NRCS of the electrically large sea surface can be calculated by small perturbation method (SPM) as [26]

(2) $$\sigma_{pq}^{SPM}({\hat{k}}_{i},{\hat{k}}_{s})=\pi k^{4}{\left|\varepsilon -1\right|}^{2}{\left|F_{pq}\right|}^{2}S(q_{⊥})\;$$

where S(·) is the sea wave spectrum corresponding to the small-scale ripples; $q_{⊥}=\left|\vec{q}\right|\sqrt{1-{\left(\hat{n}\cdot \vec{q}/\left|\vec{q}\right|\right)}^{2}}$ is the projection of $\vec{q}$ onto the triangular meshing; $\hat{n}=\left(-z_{x}\hat{x}-z_{y}\hat{y}+\hat{z}\right)/\sqrt{1+z_{x}^{2}+z_{y}^{2}}$ denotes the unit normal vector of the triangle element; $z_{x}$ and $z_{y}$ denote the slope of the triangular meshing along the *x* and *y* directions, respectively.

For co-polarization, the coefficient matrix $F_{pq}$ in Equation (2) can be expressed as

(3) $$F_{vv}=\frac{1}{\varepsilon }[1+R_{v}(\theta_{i})][1+R_{v}(\theta_{s})]\sin\theta_{i}\sin\theta_{s}-[1-R_{v}(\theta_{i})][1-R_{v}(\theta_{s})]\cos\theta_{i}\cos\theta_{s}\cos\phi_{s}\;$$

(4) $$F_{vh}=[1-R_{v}(\theta_{i})][1+R_{h}(\theta_{s})]\cos\theta_{i}\sin\phi_{s}\;$$

(5) $$F_{hv}=[1+R_{h}(\theta_{i})][1-R_{v}(\theta_{s})]\cos\theta_{s}\sin\phi_{s}\;$$

(6) $$F_{hh}=[1+R_{h}(\theta_{i})][1+R_{h}(\theta_{s})]\cos\phi_{s}\;$$

where ε is the relative permittivity of sea surface; $R_{h}$, and $R_{v}$ correspond to Fresnel coefficients in *h* (horizontal) and *v* (vertical) polarizations, respectively; $\theta_{i}$, and $\theta_{s}$ represent the global incident and scattering angle, respectively; $\phi_{i}$, and $\phi_{s}$ denote the global incident and scattering azimuth angle, respectively, as shown in Figure 1. 

The ripples which contribute to the EM scattering are modulated by large-scale gravity waves, as shown in Figure 1. Therefore, The NRCS can be calculated based on the local small perturbation method with local bi-static angles (${\theta^{\prime }}_{i}$,${\theta^{\prime }}_{s}$,${\phi^{\prime }}_{i}$,${\phi^{\prime }}_{s}$). Thus, the Equation (4) can be re-written in local coordinates as

(7) $$\sigma_{pq}^{TSM}({\hat{k}}_{i},{\hat{k}}_{s})=\pi k^{4}{\left|\varepsilon -1\right|}^{2}{\left|{\tilde{F}}_{pq}\right|}^{2}S(q_{⊥})\;$$

(8) $$\left[\begin{array}{l} {\tilde{F}}_{vv}\;{\tilde{F}}_{vh}\\ {\tilde{F}}_{hv}\;{\tilde{F}}_{hh} \end{array}\right]=\left[\begin{array}{l} {\hat{v^{\prime }}}_{i}\cdot {\hat{v}}_{i}\;{{\hat{v}}^{\prime }}_{i}\cdot {\hat{h}}_{i}\\ {{\hat{h}}^{\prime }}_{i}\cdot {\hat{v}}_{i}\;{{\hat{h}}^{\prime }}_{i}\cdot {\hat{h}}_{i} \end{array}\right]\left[\begin{array}{l} F_{vv}\;F_{vh}\\ F_{hv}\;F_{hh} \end{array}\right]\left[\begin{array}{l} {\hat{v^{\prime }}}_{s}\cdot {\hat{v}}_{s}\;{{\hat{v}}^{\prime }}_{s}\cdot {\hat{h}}_{s}\\ {{\hat{h}}^{\prime }}_{s}\cdot {\hat{v}}_{s}\;{{\hat{h}}^{\prime }}_{s}\cdot {\hat{h}}_{s} \end{array}\right]\;$$

where ${\hat{v}}_{i}$, ${\hat{h}}_{i}$, ${{\hat{v}}^{\prime }}_{i}$, and ${{\hat{h}}^{\prime }}_{i}$ indicate the unit incident polarization in global and local coordinates, respectively; ${\hat{v}}_{s}$, ${\hat{h}}_{s}$, ${{\hat{v}}^{\prime }}_{s}$, and ${{\hat{h}}^{\prime }}_{s}$ indicate the unit scattering polarization in global and local coordinates, respectively.

### 2.3. Slope-Deterministic Composite Scattering Model (SDCSM)

For different radar frequencies at diverse incident angles, EM backscattering echoes from diverse triangular meshing on the electrically large sea surface correspond to different scattering mechanisms. When the incident angle is close to the specular reflection region, The SDKAM [6,7] is adequate for specular reflection caused by large-scale gravity waves. However, as the incident angle increases, the number of triangular meshing corresponding to the Bragg scattering mechanism is gradually increasing, which leads to SDKAM insufficient for calculating NRCS. In this case, the TSM [11,12] can be utilized to calculate the NRCS from sea surface. Therefore, it is sensible to take both scattering mechanisms into account when calculating NRCS from an electrically large sea surface, the so-called slope-deterministic composite scattering model (SDCSM) [13]. The NRCS of EM scattering echoes then can be written as

(9) $$\sigma_{pq}^{SDCSM}=\sum_{i=1}^{M}\sum_{j=1}^{N}\left[(\sigma_{pq,ij}^{SDTSM}+\sigma_{pq,ij}^{SDKAM})\Delta x\Delta y\right]/A\;$$

where *M* and *N* represent sample points of the sea surface model in the *x* and *y* directions, respectively; Δx and Δy correspond to the spatial step of the sea surface model in the *x* and *y* directions, respectively; *A* is the area of the electrically large sea surface.

**Figure 2:**
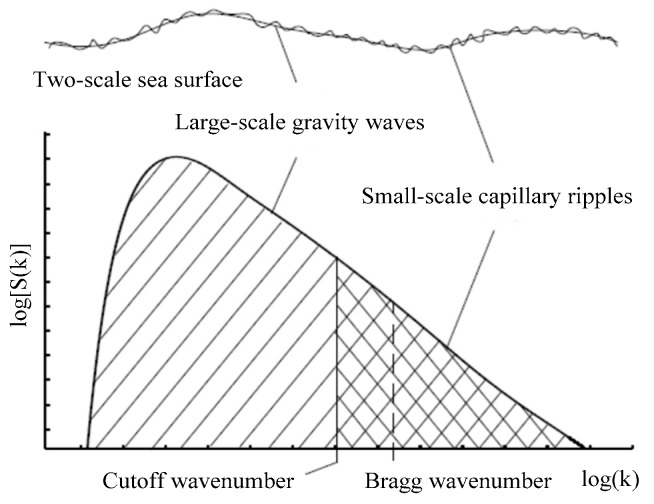
Sea wave spectrum *S*(*k*) versus the wavenumber.

The cutoff wavenumber $k_{cut}$ is exploited to distinguish the quasi-specular scattering mechanism caused by large-scale gravity waves from the Bragg scattering caused by small-scale ripples, as shown in Figure 2. When $q_{⊥}$ is less than the cutoff number, the NRCS of EM backscattering echoes from an individual facet is responsible for the quasi-specular scattering mechanism which can be well calculated by SDKAM in Equation (1). When $q_{⊥}$ is greater than the cutoff wavenumber, the NRCS of EM backscattering echoes from an individual facet is responsible for the Bragg scattering mechanism which can be well calculated by SDTSM in Equation (7). Then the NRCS of EM scattering echoes can be calculated as the superposition of the contribution from diverse scattering facets on the electrically large sea surface based on Equation (9). In this paper, $k_{cut}=k/3$ is adopted in our model according to the results in [27] when the sea spectrum is Efouhaily spectrum [28]. 

**Figure 3:**
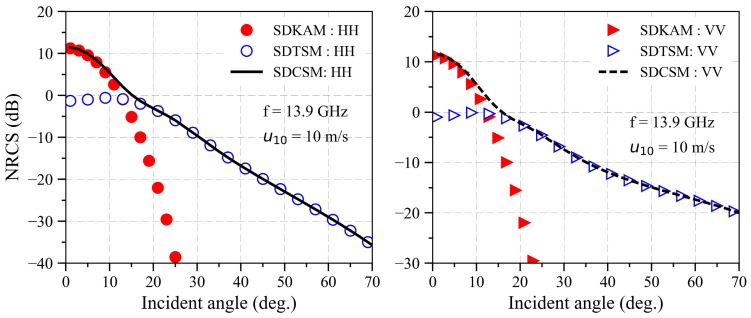
Normalized radar cross section (NRCS) of EM backscattering echoes from an electrically large sea surface versus the incident angles based on the Slope-Deterministic Kirchhoff Approximation Model (SDKAM), Slope-Deterministic Two-Scale Model (SDTSM), and slope-deterministic composite scattering model (SDCSM) when cutoff number is equal to k/3.

For backscattering NRCS computation, $\theta_{i}=\theta_{s}$, $\phi_{i}=0{}^{\circ}$ and $\phi_{s}=180{}^{\circ}$. Figure 3 displays the NRCS of EM backscattering echoes from an electrically large sea surface based on SDKAM, SDTSM, and SDCSN, respectively. The frequency is equal to 13.9 GHz, the wind speed $u_{10}=10\;m/s$, the wind direction is downwind. The size of electrically large sea surface is 204.9 m×204.9 m, the spatial step is equal to 0.1 m×0.1 m, the cutoff number $k_{cut}=k/3$, the permittivity of the sea surface is equal to (25.4047, 36.0192). The results in this paper are the average calculation over 50 samples. As shown in Figure 3, when the incident angle is close to the specular scattering region, the NRCS calculated by SDKAM is 10 dB greater than that calculated by SDTSM, indicating that the SDTSM is not suitable for calculating the specular backscattering echoes. However, when the incident angle is greater than 20 degrees, the NRCS calculated by SDKAM will decrease dramatically due to the EM backscattering echoes from sea surface being dominated by Bragg scattering caused by small-scale ripples.

**Figure 4:**
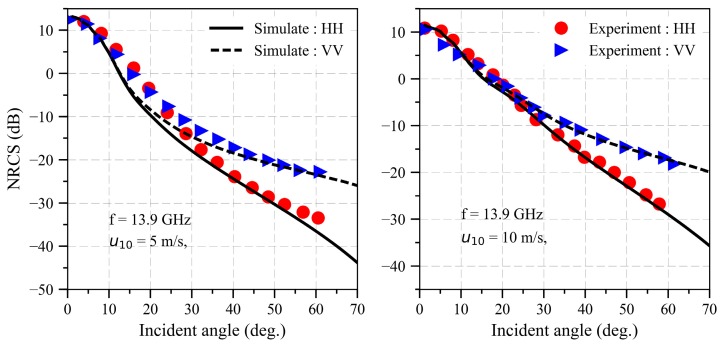
NRCS of electrically large oceanic surface compared with the experiment data when the frequency is equal to 13.9 GHz at different wind speed.

The NRCS of an electrically large sea surface compared with the experiment data [29] is illustrated in Figure 4 when the wind speeds are 5 and 10 m/s, respectively. The other parameters are the same as Figure 4. As can be seen in Figure 4, the results based on SDCSM in this paper are relatively consistent with the experiment data, which indicates that our algorithm is feasible to calculate the NRCS of EM backscattering echoes from sea surface. However, with the development of synthetic aperture radar (SAR), the resolution of the electrically large sea surface is gradually improved, which leads to the inefficiency calculation of NRCS form electrically large sea surface. Therefore, it is necessary to develop a high performance algorithm to calculate the NRCS of electrically large sea surface.

## 3. NVIDIA Tesla K80 GPU Features and GPU-Based SDCSM Implemented

In this paper, NVIDIA Tesla K80 GPU with CUDA is exploited to improve the calculation performance of NRCS from electrically large sea surface. The GPU accelerator features and the GPU-based SDCSM algorithm are detailed in this section.

### 3.1. NVIDIA Tesla K80 GPU Haredare Resource

**Figure 5:**
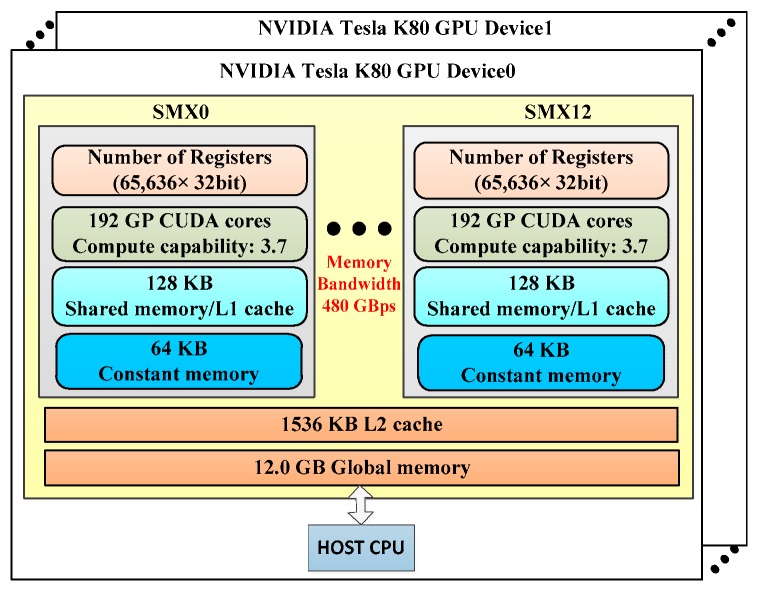
Schematic visualization of NVIDIA Tesla K80 dual-GPU accelerator features.

NVIDIA Tesla K80 is engineered to deliver superior performance with more powerful servers. It is designed to boost throughput by 5~10× with 480 GB/s aggregate memory bandwidth compared with conventional CPU counterpart. Figure 5 displays the schematic of Tesla K80 dual-GPU features. As shown in Figure 5, NVIDIA Tesla K80, with dual-GPU design features, contains 24 GB of global memory, 65,536 registers, 128 KB of shared memory, and 64 KB of constant memory. There are also 13 stream multiprocessors (SMX) in each GPU and each SMX contains 192 CUDA cores, with a total of 4992 NVIDIA CUDA cores for Tesla K80. Our sequential C algorithm is performed on the Intel(R) core (TM) i5-3450 CPU.

### 3.2. SDCSM Parallel Computing with CUDA

CUDA was introduced in 2006 by NVIDIA as an extension of conventional C program to leverage the high performance computing (HPC) capability of GPUs, which enables scientists and engineers to solve massive computational problems in a more efficient way. In this paper, an efficient GPU-based SDCSM model is developed with CUDA to improve the calculation performance of NRCS from an electrically large sea surface. The entirely parallel program consists of two components: the sequential parts performed on CPU and the parallel parts performed on GPU.

**Figure 6:**
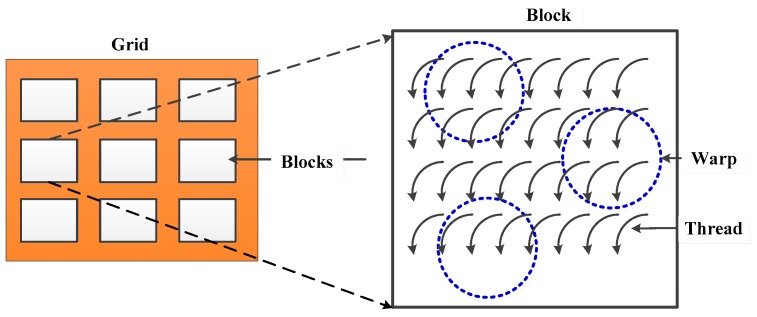
Schematic visualization of a hierarchy of thread groups.

Threads are the smallest executable unit in a CUDA program, in which a three-dimensional thread forms a thread block, and a grid is made up of a three-dimensional thread block. Figure 6 shows a hierarchy of thread groups. On the Tesla K80, each SMX schedules and performs threads concurrently in a group of 32 CUDA cores, which is usually called a warp. The maximum number of threads per multiprocessor is 2048.

The typical processing flow of the GPU-based SDCSM algorithm can be expressed as follows:

1. Initialize the size of electrically large sea surface $L_{x}\times L_{y}$, the spatial step of the sea surface Δx×Δy, the wind speed $u_{10}$, the wind direction $\phi_{w}$, the incident and scattering angles $\theta_{i}$ and $\theta_{s}$, the incident and scattering azimuth angles $\phi_{i}$ and $\phi_{s}$, the frequency f, the grid and block sizes corresponding to the CUDA program.
2. Transfer the electrically large sea surface data from the CPU to the GPU.
3. Compute the NRCS of individual triangular meshing on the electrically large sea surface independently in parallel on the GPU by all threads within a block.
4. Copy the results from the GPU back to the CPU.

In this paper, the EM backscattering echoes from different triangular meshes are de-correlation, thus the EM backscattering echoes from diverse triangular meshes on the electrically large sea surface can be calculated independently in parallel on the GPU by all threads. Each thread is responsible for calculating the EM backscattering from individual triangular meshing on the sea surface. 

## 4. Initial Parallel Implemented and Further Optimization

### 4.1. Initial Parallel Implemented

**Figure 7:**
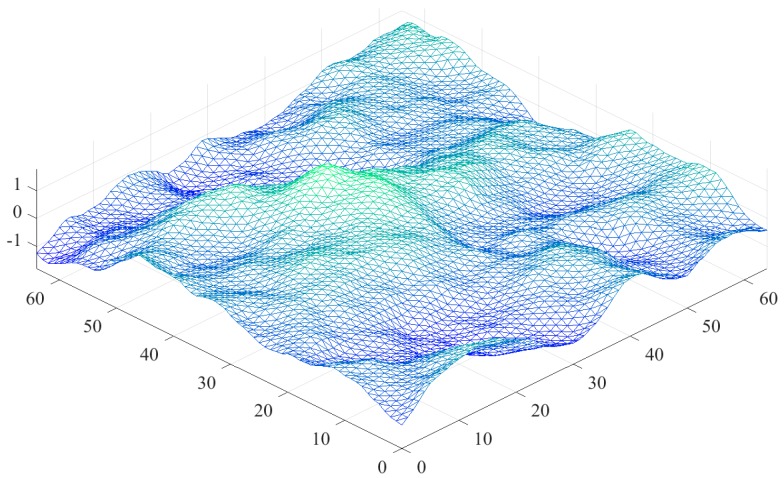
Schematic of electrically large sea surface discretized into triangular meshes.

The computational performance of a GPU-based SDCSM algorithm will be detailed in this section, and five CUDA optimization strategies are adopted to further accelerate the computation of NRCS of electrically large sea surface. The frequency f=13.9 GHz, the wind speed $u_{10}=10\;m/s$, the incident angle $\theta_{i}=60{}^{\circ}$ and $\theta_{s}=\theta_{i}$, the incident and scattering azimuth angles are 0° and $180^{0}$, respectively. Figure 7 shows the schematic of electrically large sea surface discretized into triangular meshes. The size of the electrically large sea surface is $204.9\times 204.9\;m^{2}$, the spatial step is equal to $0.1\times 0.1\;m^{2}$. Thus, there will be 8,396,802 triangular meshing when calculating the NRCS of the electrically large sea surface. The grid and block sizes are equal to 128×256 and 16×16, respectively.

The GPU-based SDCSM parallel program executed on the Tesla K80 was compiled by the nvcc 5.0 with –O3 –arch=compute_35 –code = sm_35 –Xptxas –v compiling option while the conventional serial C program executed on Intel(R) core (TM) i5-3450 CPU was compiled by g++ with –O2 compiling option. Table 1 shows the runtime and speedup for the GPU-based SDCSM parallel program compared with the conventional serial C program. As shown in Table 1, the initial GPU-based SDCSM program only takes 86.31 ms, while the CPU-based program requires 47,593.6 ms. A significant speedup of 551.4× is achieved compared with the serial C program. As described in Table 1, the most time-consuming aspect of the GPU-based TESF is the kernel execution. Therefore, optimizing that step is helpful to improve the performance of the CUDA program.

### 4.2. Further Optimization with Coalesced Global Memory Access

Threads inside each block may access data from diverse memory space each time that the kernel is called. Memory performance optimizations are particularly important for the massive parallel computation performance. When the CUDA program is launched, all threads performed on the SMXs will access the same global memory, which has the greatest access latency. For the Tesla K80, each SMXs provides an on-chip shared memory which can be accessible by all threads inside each block within one thread block with much higher bandwidth and lower latency. 

**Figure 8:**
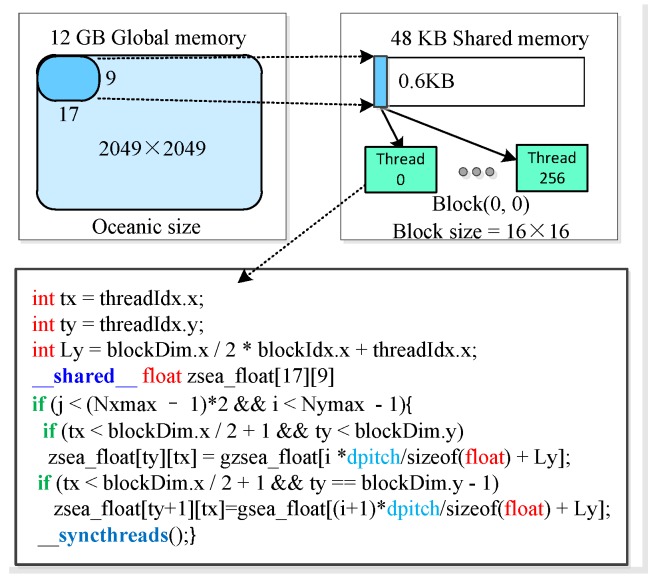
Schematic of the usage of shared memory in place of global memory for data transfers between global memory and the device.

In order to achieve high memory bandwidth, shared memory is utilized to coalesce cache global memory, thus reducing the number of accesses to the global in our parallel program. By utilizing the shared memory __shared__ zsea_float [9,17], the coalesced memory access pattern enables the GPU to coalesce groups of reads of one sub-surface data items on the electrically large sea surface into one operation, as illustrated in Figure 8. The runtime and speedup for the GPU-based SDCSM program with and without coalesced access optimization are displayed in Table 2. With coalesced accesses optimization, the number of accesses to global memory is reduced from 41,943,040 to 5,013,504 and a significant speedup of 566.4× has been achieved.

### 4.3. Further Optimization with Constant Memory

**Figure 9:**
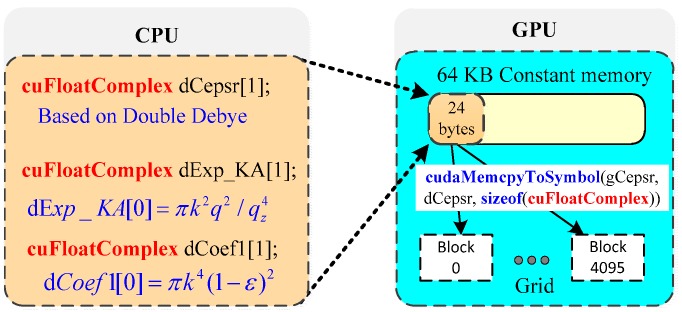
Schematic of the usage of constant memory for avoiding repeated calculations during the kernel execution.

In our CUDA program, all threads within a grid utilize the same parameters ε, $\pi k^{2}{\left|\vec{q}\right|}^{2}/q_{z}^{4}$ in Equation (1), and $\pi k^{4}{\left(1-\varepsilon \right)}^{2}$ in Equation (7) to conduct the same computation on diverse triangular meshing data. For Tesla K80 GPU, all threads call the same 64 KB constant memory storage. Figure 9 shows the usage of the constant memory for avoiding repeated calculations when the kernel is called. As shown in Figure 9, the same 24 bytes of coefficients reside in constant memory. When all threads inside a SMX access the same address, the constant cache access will be as fast as a register access.

Table 3 shows the runtime and speedup for the GPU-based SDCSM program with and without constant memory access optimization. With the constant memory optimization, the total runtime is reduced from 84.03 ms to 82.56 ms and a significant speedup of 576.5× is achieved.

### 4.4. Further Optimization with Fast Math Compiler Option

For single-precision floating-point operations performed on the device, the nvcc compiler provides an option –use_fast_math which coerces all functions in the CUDA program to compiler to CUDA intrinsic functions. Intrinsic functions are compiled with fewer instructions and greater throughput compared with their equivalent standard counterpart. As a result, the CUDA program with fast math compiler option can achieve more aggressive optimization with less accuracy. 

**Figure 10:**
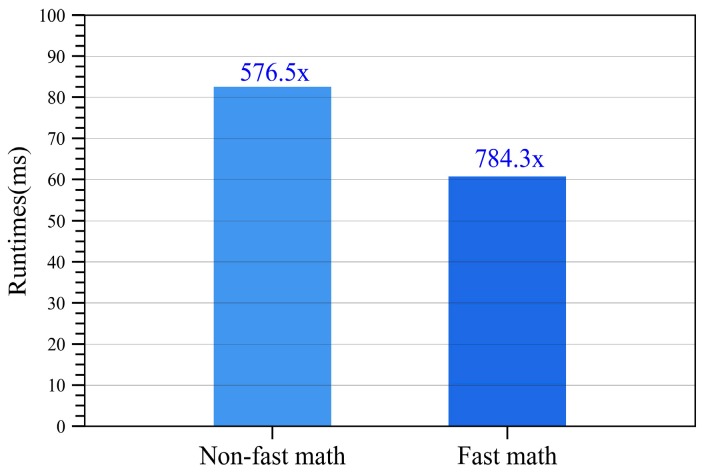
Runtime and speedup for the GPU-based SDCSM program with fast math optimization.

The runtime and speedup for the GPU-based SDCSM program with fast math compiler option is provided in Figure 10. After utilizing the –use_fast_math compiler option, the runtime for the CUDA program is reduced from 82.56 ms to 60.68 ms, resulting in an acceleration from 576.5× to 784.3×. 

It is advisable to exploit the fast math option whenever speedup is more important than precision. In order to verify the correctness of the outcomes with the –use_fast_math compiler option, the mean absolute error (MAE) and MEA/mean are exploited to ensure that the results of the parallel program are consistent with those of serial program. Table 4 illustrates the results of MAE and MAE/mean with and without the –use_fast_math compiler option. As shown in Table 4, the result of MAE and MAE/mean with the –use_fast_math compiler option is slighter larger than that without –use_fast_math compiler option, indicating that the compiler option with the fast math option has litter effect on the result correctness.

### 4.5. Further Optimization with Asynchronous Data Transfer (ADT)

**Figure 11:**
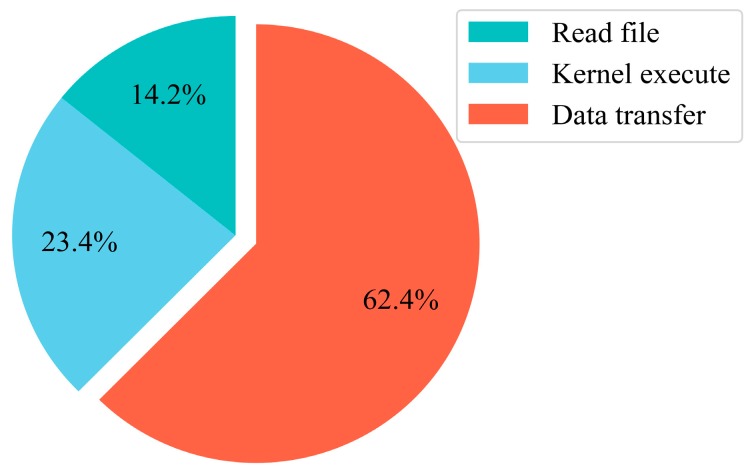
Description of the percentage of GPU runtime consumed by different GPU operations after using the fast math compiler option.

The percentages of GPU runtime consumed by different GPU operations after using the fast math compiler option are shown in Figure 11. As can been seen, the data transfer time is from 43.62% shown in Table 1 up to 62.1% in Figure 10. Therefore, reducing the data transmission time between the host and the device will effectively improve the computing performance. 

In order to hide the data transmission time, asynchronous data transfer (ADT) was utilized, which can perform data transfer between host and device memories and kernel execution simultaneously. The Tesla K80 GPU provides two copy engines, namely, a host-to-device engine and a device-to-host engine, allowing one data transfer from the host to the device, one kernel execution, and one data transfer from the device to the host to overlap. Data transfer and kernel execution perform simultaneously through streams, which can be seen as a sequence of commands that perform on the GPU in order.

**Figure 12:**
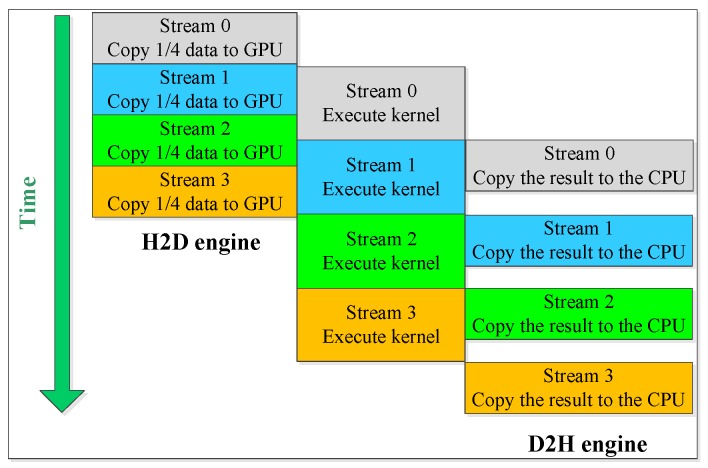
Description of the asynchronous data transfer (ADT) for GPU-based SDCSM program.

**Figure 13:**
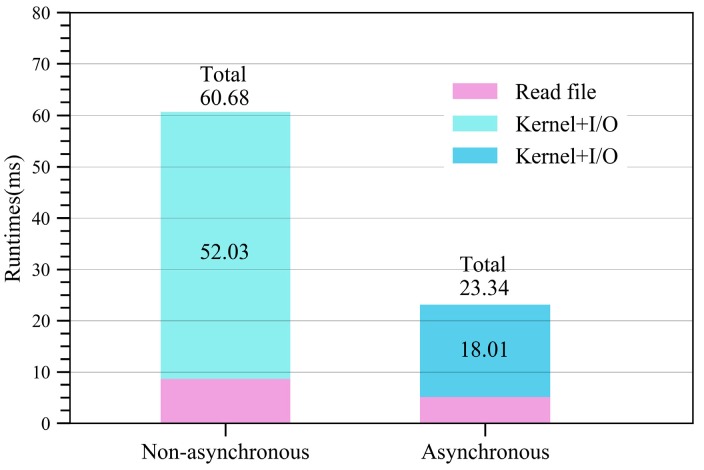
Runtime for the GPU-based SDCSM program with and without asynchronous data transfer (ADT) optimization.

Figure 12 shows the asynchronous data transfer (ADT) for our CUDA program executed on NVIDIA Tesla K80. In this paper, four streams are utilized to calculate the NRCS from the electrically large sea surface. Figure 13 shows the runtime for the GPU-based SDCSM program with and without asynchronous data transfer (ADT) optimization. As shown in Figure 13, the running time of the parallel program decreased obviously from 60.68 ms to 23.34 ms, and a significant speedup of 2031.9× can be achieved with asynchronous data transfer (ADT) optimization, as shown in Table 5. 

**Figure 14:**
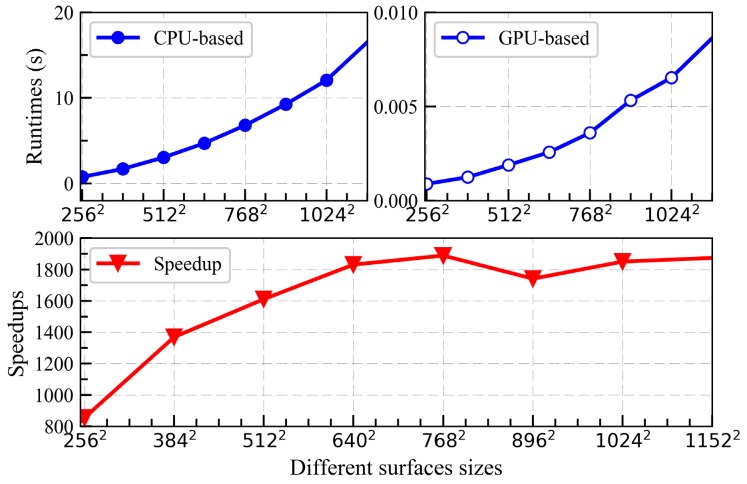
Runtime and speedup for the GPU-based SDCSM program for different surface sizes.

**Figure 15:**
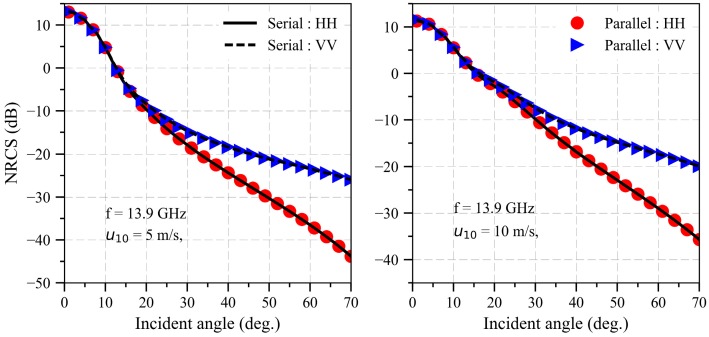
NRCS results of parallel program compared with that of serial program.

Figure 14 illustrates the speedup and runtime for different sizes of the electrically large sea surface (0.1 m). As shown in Figure 14, the speedup tends to be stable with the increase of sea surface size, which implies that the massively parallel computing performance of the GPU has been fully utilized in this paper. Figure 15 shows the NRCS results of parallel program compared with that of the serial program when the frequency is equal to 13.9 GHz at different wind speeds. As shown in Figure 15, the results of parallel are consistent with that of serial programs, which indicates that the GPU-based SDCSM algorithm developed in this paper is feasible to calculate the EM backscattering NRCS from the electrically large sea surface. A significant speedup of 2039.1× has been achieved compared with conventional serial C program, implying that the EM backscattering NRCS calculated more than 1 h now can be realized within 2 s.

## 5. Conclusions

In this paper, a slope-deterministic composite scattering model (SDCSM) was exploited to calculate the NRCS of the EM backscattering echoes from the electrically large sea surface. Both the quasi-specular backscattering caused by large-scale gravity waves and the Bragg backscattering caused by small-scale ripples are taken into account for calculating the NRCS. Moreover, the NVIDIA Tesla K80 with CUDA has been utilized to accelerate the calculation of NRCS from electrically large sea surface. Compared to the conventional serial C program, our parallel program has significantly improved the performance of the NRCS calculation and a significant speedup of 2104.2× has been achieved. Four optimization strategies have been adopted in our GPU-based SDCSM parallel program. First, coalesced global memory access was used to reduce access to global memory. Following this procedure, constant memory is exploited to reduce repetitive computations in the CUDA program. The compiler option with –use_fast_math was adopted to further improve the computational performance and the correctness of outcomes is verified to ensure the results of the parallel program are consistent with that of the serial program. Then, in order to hide the data transmission time between the host and the device, asynchronous data transfer (ADT) was exploited to further improve the computational performance of the GPU-based SDCSM parallel program. The proposed GPU-based SDCSM parallel program only calculates the NRCS of the EM backscattering under low sea state (<10 m/s). However, for high sea state (≥10 m/s), the electrically large sea surface will be covered with whitecaps. Future work will take the volume scattering into account to calculate the NRCS of the EM backscattering echoes from electrically large sea surface.

## Figures and Tables

**Table 1:** Runtime and speedup for the graphics processing units (GPU)-based SDCSM program compared with conventional serial C program.

	Total Time	Read File	Execution Time	I/O
Serial program (ms)	47,593.6	--	--	--
Parallel program (ms)	86.31	8.663	39.99	37.657
speedup	551.4×	--	--	--

**Table 2:** Runtime and speedup for the GPU-based SDCSM program with coalesced global memory access optimization.

	CPU Runtime (ms)	GPU-Runtime (ms)	Speedup
Serial program	47,593.6	--	--
Initial Parallel program		86.31	551.4x
Utilizing shared memory		84.03	566.4x

**Table 3:** Runtime and speedup for the GPU-based SDCSM program with constant memory access optimization.

	CPU Runtime (ms)	GPU-Runtime (ms)	Speedup
Serial program	47,593.6	--	--
Non-optimized		84.03	566.4×
Optimized		82.56	576.5×

**Table 4:** The results of mean absolute error (MAE) and MAE/mean with and without the *–use_fast_math* compiler option.

Variable	Variable Description	Non-Fast Math		Fast Math	
		MAE	MAE/Mean	MAE	MAE/Mean
$\sigma_{hh}^{SDCSM}$	The NRCS for HH polarization	$1.014\times 10^{-11}$	$1.590\times 10^{-6}$	$1.226\times 10^{-11}$	$1.923\times 10^{-6}$
$\sigma_{vv}^{SDCSM}$	The NRCS for VV polarization	$1.261\times 10^{-10}$	$1.352\times 10^{-6}$	$1.352\times 10^{-10}$	$1.451\times 10^{-6}$

**Table 5:** Runtime and speedup for the GPU-based SDCSM program with asynchronous data transfer (ADT) optimization.

	CPU Runtime (ms)	GPU-Runtime (ms)	Speedup
Serial program	47,593.6	--	--
Non-optimized		60.68	784.3×
Optimized		23.34	2039.1×
